# Supplementary material for: Microbial Diversity and Characteristic Quality Formation of Qingzhuan Tea as Revealed by Metagenomic and Metabolomic Analysis during Pile Fermentation
Source: Foods. 2023 Sep 22;12(19):3537. doi: 10.3390/foods12193537 (PMC10572444; doi:10.3390/foods12193537)
Supplement: Supplementary file 1 [file foods-12-03537-s001.zip › Table S1.pdf]

**Table S1.** The gradient elution conditions of liquid chromatography and mass spectrometry.

| Time(min) | Flow（mL/min) | A% (water+0.1%formic acid） | B% ( acetonitrile + 0.1% formic acid） |
|---|---|---|---|
| 0 | 0.3 | 95 | 5 |
| 2 | 0.3 | 60 | 40 |
| 7 | 0.3 | 20 | 80 |
| 11 | 0.3 | 5 | 95 |
| 15 | 0.3 | 5 | 95 |
| 15.5 | 0.3 | 95 | 5 |
| 20 | 0.3 | 95 | 5 |
