# Supplementary figures and images for: Microbial Diversity and Characteristic Quality Formation of Qingzhuan Tea as Revealed by Metagenomic and Metabolomic Analysis during Pile Fermentation
Source: Foods. 2023 Sep 22;12(19):3537. doi: 10.3390/foods12193537 (PMC10572444; doi:10.3390/foods12193537)

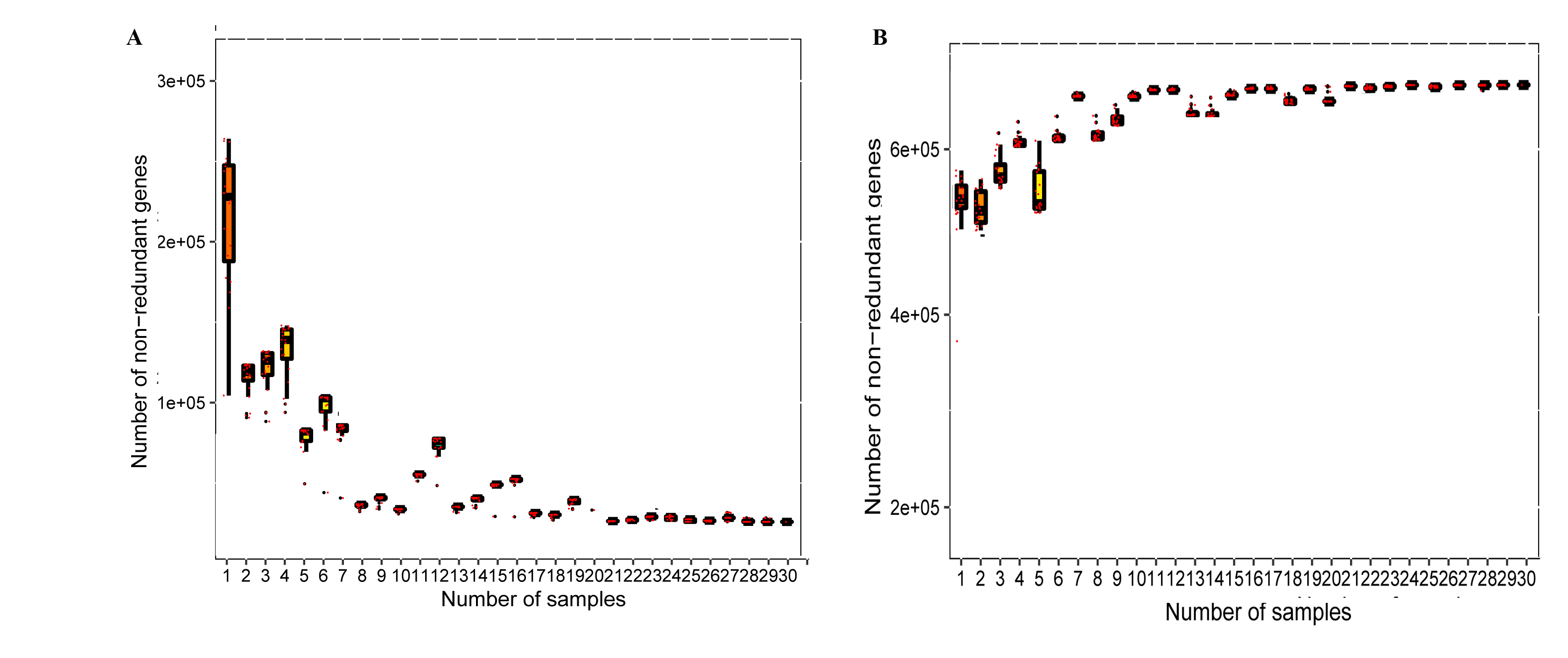

Supplement: Supplementary file 1 [file foods-12-03537-s001.zip › Figure S2.tif]

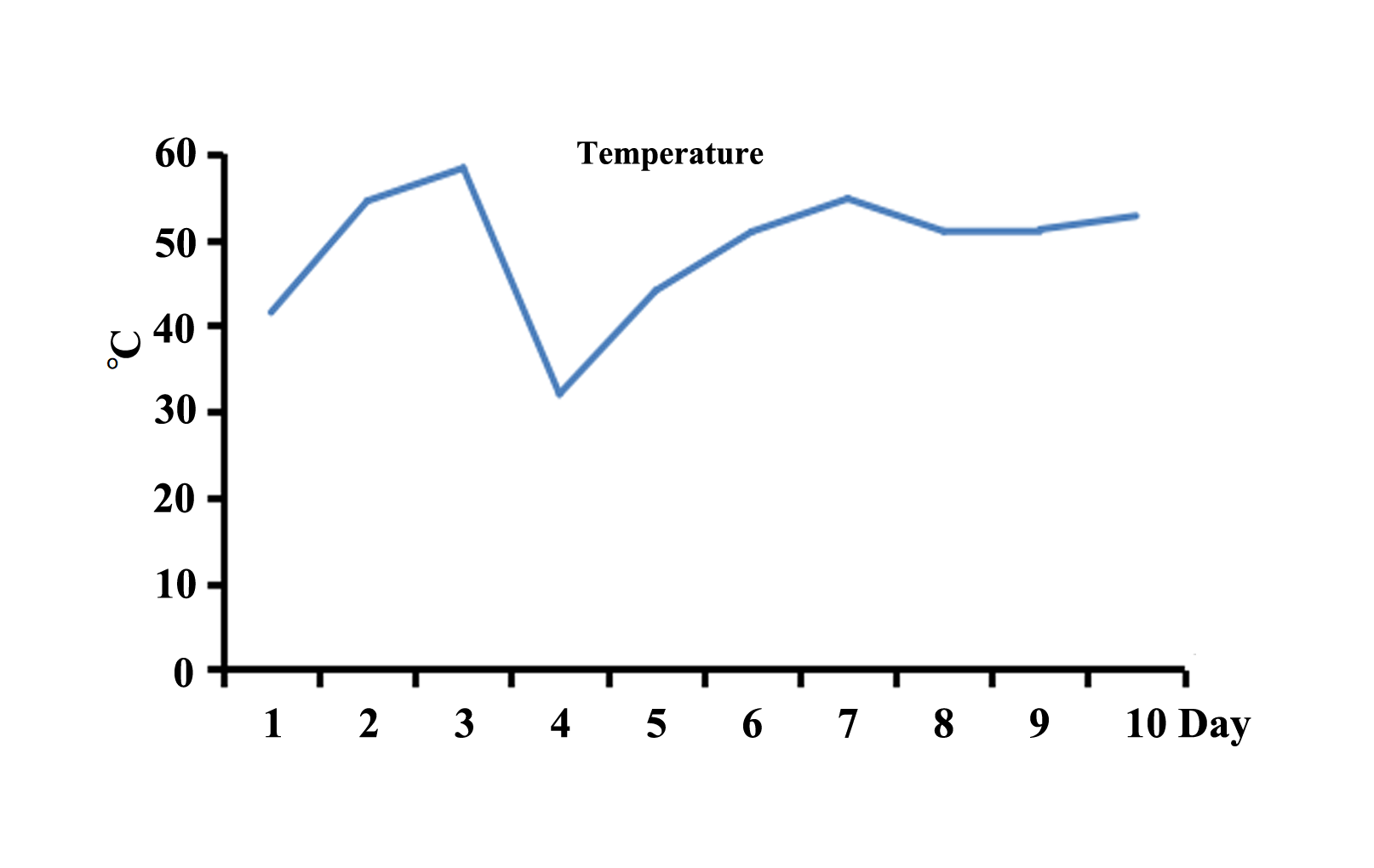

Supplement: Supplementary file 1 [file foods-12-03537-s001.zip › foods-2595641-Figure S1.tif]
